# A new polymorph of *N*-(prop-2-yn­yl)tricyclo­[3.3.1.1^3,7^]decane-1-carbox­amide

**DOI:** 10.1107/S1600536808021466

**Published:** 2008-07-16

**Authors:** Wolfgang Frey, Stefanie Schetter, Frank Rominger, A. Stephen K. Hashmi

**Affiliations:** a Institut für Organische Chemie, Universität Stuttgart, Pfaffenwaldring 55, 70569 Stuttgart, Germany; b Organisch-Chemisches Institut, Universität Heidelberg, Im Neuenheimer Feld 270, D-69120 Heidelberg, 70569 Stuttgart, Germany

## Abstract

The alkynyl bond of the title compound, C_14_H_19_NO, has a length of 1.170 (5) Å. The amide function shows a *trans* conformation with respect to the carbonyl group characterized by the torsion angle O—C—N—H of −176 (2)°. There is an inter­molecular N—H⋯O hydrogen bond between the amide function and the carbonyl group. In addition, weak inter­molecular hydrogen bonds stabilize the crystal structure. A comparison with a polymorphic structure shows conformational differences with regard to the orientation of the carbonyl groups with respect to the adamantyl group [O—C—C—C = 96.2 (3)° in the title compound and 123.7 (2)° in the polymorph] and the orientations of the propargyl groups in relation to the carbonyl groups [O—C—C—C = −87.7 (3) and −58.7 (2)°, respectively].

## Related literature

For the monoclinic polymorph, see: Hashmi *et al.* (2004 ▶). For gold catalysis research, see: Hashmi (2003 ▶, 2004 ▶, 2005 ▶, 2007 ▶); Hashmi & Hutchings (2006 ▶); Hashmi, Frost & Bats (2000 ▶); Hashmi, Schwarz *et al.* (2000 ▶); Hashmi *et al.* (2006 ▶). For the synthesis of heterocyclic compounds, see: Milton *et al.* (2004 ▶).
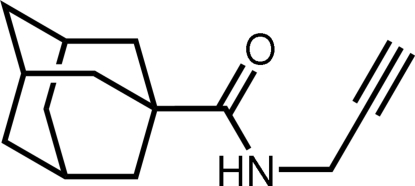


## Experimental

### 

#### Crystal data

C_14_H_19_NO
*M* *_r_* = 217.30
Orthorhombic, 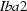

*a* = 9.862 (2) Å
*b* = 28.095 (5) Å
*c* = 8.664 (3) Å
*V* = 2400.4 (10) Å^3^
*Z* = 8
Mo *K*α radiation
μ = 0.07 mm^−1^
*T* = 293 (2) K
0.9 × 0.4 × 0.1 mm

#### Data collection

Nicolet P3 diffractometer
Absorption correction: none
13418 measured reflections
1853 independent reflections
1442 reflections with *I* > 2σ(*I*)
*R*_int_ = 0.084
3 standard reflections every 50 reflections intensity decay: 2%

#### Refinement

*R*[*F* ^2^ > 2σ(*F* ^2^)] = 0.061
*wR*(*F* ^2^) = 0.130
*S* = 1.09
1853 reflections
150 parameters
1 restraint
H atoms treated by a mixture of independent and constrained refinement
Δρ_max_ = 0.21 e Å^−3^
Δρ_min_ = −0.14 e Å^−3^

### 

Data collection: *P3/PC Software* (Siemens, 1991 ▶); cell refinement: *P3/PC Software*; data reduction: *XDISK* in *SHELXTL-Plus* (Sheldrick, 2008 ▶); program(s) used to solve structure: *SHELXS97* (Sheldrick, 2008 ▶); program(s) used to refine structure: *SHELXL97* (Sheldrick, 2008 ▶); molecular graphics: *XP* in *SHELXTL-Plus*; software used to prepare material for publication: *SHELXL97* and *PLATON* (Spek, 2003 ▶).

## Supplementary Material

Crystal structure: contains datablocks I, global. DOI: 10.1107/S1600536808021466/bt2731sup1.cif

Structure factors: contains datablocks I. DOI: 10.1107/S1600536808021466/bt2731Isup2.hkl

Additional supplementary materials:  crystallographic information; 3D view; checkCIF report

## Figures and Tables

**Table 1:** Hydrogen-bond geometry (Å, °) *X*1 is the midpoint of the alkynyl bond.

*D*—H⋯*A*	*D*—H	H⋯*A*	*D*⋯*A*	*D*—H⋯*A*
N1—H1*A*⋯O1^i^	0.86 (3)	2.16 (4)	2.984 (4)	161 (3)
C1—H1⋯O1^ii^	0.93	2.41	3.188 (5)	141
C10—H10*B*⋯O1^i^	0.97	2.57	3.515 (4)	164
C3—H3*A*⋯*X*1^iii^	0.97	2.93	3.833	157
C3—H3*B*⋯*X*1^iv^	0.97	2.93	3.813	151
C6—H6*A*⋯*X*1^v^	0.97	2.81	3.689	151

Symmetry codes: (i) 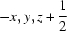; (ii) 
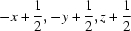
; (iii) 
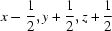
; (iv) 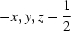; (v) 
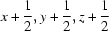
.

**Fig. 1.**
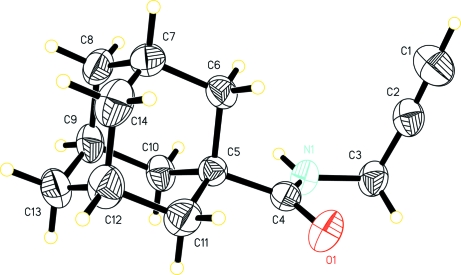
Perspective view of the title compound with atom numbering. Displacement ellipsoids are at the 50% probability level.

**Fig. 2.**
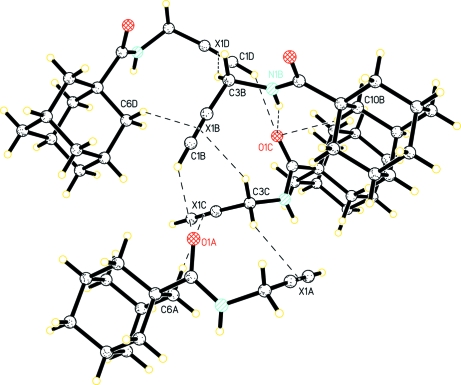
Detailed view of the intermolecular hydrogen bond interactions. (X1A - X1D are the centers of the alkynyl bond)

**Fig. 3.**
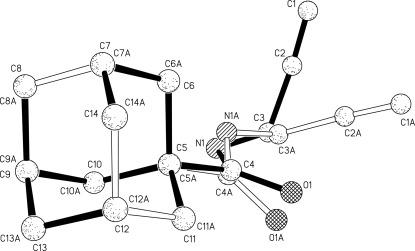
Superposition plot of the title compound (bold bonds) and the formerly reported polymorphic structure (Mol. A, open bonds). For better insight plot without Hydrogen atoms.

**Fig. 4.**
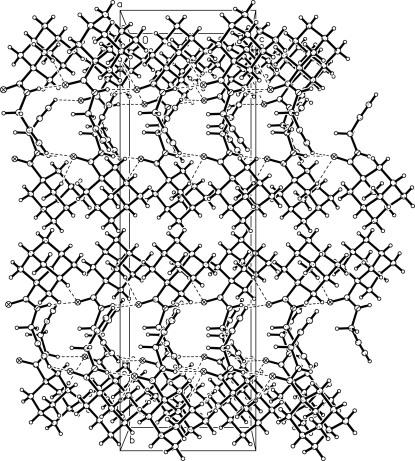
Cell Plot of the title compound (bc-view, inclusive hydrogen atoms)

## supplementary crystallographic information

### Comment

Initiated by our early work on gold-catalyzed additions of nucleophiles to allenes (Hashmi *et al.*,  2000*a*; Hashmi *et al.*,  2000*b*) and alkynes (Hashmi & Frost *et al.*, 2000) the investigation of the synthetic potential of propargylic carboxamides in gold-catalyzed reactions (Hashmi, 2007) revealed that they can be excellent precursors for the formation of oxazoles (Hashmi, Weyrauch, Frey & Bats, 2004) and alkylidene oxazolines (Hashmi & Rudolph *et al.*, 2006) in highly selective reactions under mild conditions. As one of the substrates with bulky, sterically demanding substiutents which documented the broad scope of the reaction, the title compound was prepared.

The title compound (Fig. 1) crystallizes with one molecule in the asymmetric unit of the space group Iba2. The alkynyl bond was clearly identified by the distance of 1.170 (5) Å between the carbon atoms C1 and C2. As expected, the N1—H1A amide function shows a *trans* conformation concerning to the carbonyl group C4=O1 indicated by the torsion angle O1—C4—N1—H1A of -176 (2)°. The crystal packing (Fig. 4) is stabilized by a number of intermolecular hydrogen bond contacts (Fig. 2). A strong intermolecular hydrogen bond works between the amide function N1—H1A as donor and the oxygen O1 of the carbonyl group as acceptor with a H1A···O1 distance of 2.16 Å and an angle N1—H1A···O1 of 161°. The oxygen O1 of the carbonyl function is also an acceptor in more weak interactions, where the alkynyl moiety C1—H1 and the methylen group C10—H10B of the adamantyl system works as donors. The H1···O1 distance is 2.41 Å and the H10B···O1 distance is 2.57 Å respectively. The center of the alkynyl bond X1 works also as acceptor of weak hydrogen bond interactions, where the methylen group of the propargyl moiety C3—H3A and C3—H3B are the donors. The distances of H3A···X1 and H3B···X1 are both 2.93 Å. X1 is also the acceptor of an weak interaction including the methylen group C6—H6A of the adamantyl moiety with a distance H6A···X1 of 2.81 Å (see Table). Hashmi & Weyrauch *et al.*, 2004, reported about a polymorphic structure (further abbreviated as Mol.A) of the title compound crystallized in space group *C*2/*c*, which was also crystallized by slow diffusion of petrol ether into a solution of dichloromethane. To get more insight of structural differences the carbon atoms of the adamantyl moieties of both structures were superimposed yielded in an optimal fit with a weighted r.m.s. of 0.0061 Å (Fig. 3, bold bonds shows title compound). The orientations of the carbonyl functions C4=O1 in relation to the adamantyl moiety are quite different characterized by the torsion angle O1—C4—C5—C6 of 96.2 (3)° and 123.7 (2)° (Mol.A). The orientations of the propargyl groups in relation to the carbonyl functions differs also significant indicated by the Newman projection along the carbons C3 and C4 with torsion angles O1—C4—C3—C2 of -87.7 (3)° and -58.7 (2)° (Mol.A) respectively.

### Experimental

The title compound was prepared by the reaction of adamantane-1-carboxylic acid chloride with propargyl amine in dichloromethane at 0–20 °C using a 2 mol% of 4-*N*,*N*-dimethylaminopyridine as a catalyst in 76% yield as described previously (Hashmi, Weyrauch, Frey & Bats, 2004). Crystals were grown by slow diffusion of petrol ether into a solution of dichloromethane.

### Refinement

H atoms were located in difference fourier map, but refined with fixed individual displacement parameters [U(H) = 1.2 *U*_eq_(C)] using a riding model with C—H ranging from 0.93 to 0.98 Å. H1A of the amide function was refined free with individual displacement parameters, because of its relevance for the geometry of the hydrogen bond interaction.

### Crystal data

**Table:**

C_14_H_19_NO	*F*_000_ = 944
*M**_r_* = 217.30	*D*_x_ = 1.203 Mg m^−^^3^
Orthorhombic, *I**b**a*2	Mo *K*α radiation λ = 0.71073 Å
Hall symbol: I 2 -2c	Cell parameters from 32 reflections
*a* = 9.862 (2) Å	θ = 9–13º
*b* = 28.095 (5) Å	µ = 0.08 mm^−^^1^
*c* = 8.664 (3) Å	*T* = 293 (2) K
*V* = 2400.4 (10) Å^3^	Plates, colourless
*Z* = 8	0.9 × 0.4 × 0.1 mm

### Data collection

**Table:**

Nicolet P3 diffractometer	*R*_int_ = 0.084
Radiation source: fine-focus sealed tube	θ_max_ = 30.0º
Monochromator: graphite	θ_min_ = 2.2º
*T* = 293(2) K	*h* = −13→13
Wyckoff ccan scans	*k* = −39→39
Absorption correction: none	*l* = −12→12
13418 measured reflections	3 standard reflections
1853 independent reflections	every 50 reflections
1442 reflections with *I* > 2σ(*I*)	intensity decay: 2%

### Refinement

**Table:**

Refinement on *F*^2^	Secondary atom site location: difference Fourier map
Least-squares matrix: full	Hydrogen site location: inferred from neighbouring sites
*R*[*F*^2^ > 2σ(*F*^2^)] = 0.061	H atoms treated by a mixture of independent and constrained refinement
*wR*(*F*^2^) = 0.130	*w* = 1/[σ^2^(*F*_o_^2^) + (0.0432*P*)^2^ + 1.5609*P*] where *P* = (*F*_o_^2^ + 2*F*_c_^2^)/3
*S* = 1.09	(Δ/σ)_max_ < 0.001
1853 reflections	Δρ_max_ = 0.21 e Å^−^^3^
150 parameters	Δρ_min_ = −0.14 e Å^−^^3^
1 restraint	Extinction correction: SHELXL97 (Sheldrick, 2008), Fc^*^=kFc[1+0.001xFc^2^λ^3^/sin(2θ)]^-1/4^
Primary atom site location: structure-invariant direct methods	Extinction coefficient: 0.0008 (7)

### Special details

**Table:**

Geometry. All e.s.d.'s (except the e.s.d. in the dihedral angle between two l.s. planes) are estimated using the full covariance matrix. The cell e.s.d.'s are taken into account individually in the estimation of e.s.d.'s in distances, angles and torsion angles; correlations between e.s.d.'s in cell parameters are only used when they are defined by crystal symmetry. An approximate (isotropic) treatment of cell e.s.d.'s is used for estimating e.s.d.'s involving l.s. planes.
Refinement. Refinement of *F*^2^ against ALL reflections. The weighted *R*-factor *wR* and goodness of fit *S* are based on *F*^2^, conventional *R*-factors *R* are based on *F*, with *F* set to zero for negative *F*^2^. The threshold expression of *F*^2^ > σ(*F*^2^) is used only for calculating *R*-factors(gt) *etc*. and is not relevant to the choice of reflections for refinement. *R*-factors based on *F*^2^ are statistically about twice as large as those based on *F*, and *R*- factors based on ALL data will be even larger.

### Fractional atomic coordinates and isotropic or equivalent isotropic displacement parameters (Å^2^)

**Table:**

	*x*	*y*	*z*	*U*_iso_*/*U*_eq_	
O1	0.1418 (2)	0.17494 (8)	0.6152 (3)	0.0516 (6)	
N1	0.0006 (3)	0.18653 (8)	0.8136 (3)	0.0417 (5)	
H1A	−0.023 (3)	0.1792 (9)	0.906 (4)	0.030 (8)*	
C1	0.1177 (4)	0.29834 (13)	0.8851 (6)	0.0648 (11)	
H1	0.1746	0.3204	0.9322	0.078*	
C2	0.0462 (3)	0.27061 (10)	0.8258 (5)	0.0471 (7)	
C3	−0.0418 (3)	0.23362 (9)	0.7609 (4)	0.0466 (7)	
H3A	−0.1348	0.2394	0.7923	0.056*	
H3B	−0.0380	0.2349	0.6491	0.056*	
C4	0.1019 (3)	0.16321 (9)	0.7436 (3)	0.0362 (6)	
C5	0.1706 (2)	0.12334 (9)	0.8342 (3)	0.0321 (5)	
C6	0.2719 (3)	0.14739 (9)	0.9446 (4)	0.0408 (7)	
H6A	0.3346	0.1669	0.8858	0.049*	
H6B	0.2233	0.1680	1.0155	0.049*	
C7	0.3511 (3)	0.10992 (10)	1.0360 (4)	0.0454 (7)	
H7	0.4157	0.1258	1.1049	0.055*	
C8	0.2518 (3)	0.08042 (11)	1.1315 (4)	0.0464 (7)	
H8A	0.3010	0.0570	1.1916	0.056*	
H8B	0.2028	0.1009	1.2021	0.056*	
C9	0.1523 (3)	0.05549 (9)	1.0236 (4)	0.0441 (7)	
H9	0.0889	0.0363	1.0847	0.053*	
C10	0.0723 (3)	0.09276 (9)	0.9302 (4)	0.0413 (6)	
H10A	0.0087	0.0768	0.8622	0.050*	
H10B	0.0213	0.1130	0.9999	0.050*	
C11	0.2495 (3)	0.09040 (11)	0.7245 (4)	0.0482 (8)	
H11A	0.1868	0.0748	0.6546	0.058*	
H11B	0.3124	0.1091	0.6633	0.058*	
C12	0.3275 (3)	0.05301 (11)	0.8169 (4)	0.0524 (8)	
H12	0.3773	0.0323	0.7458	0.063*	
C13	0.2292 (3)	0.02307 (10)	0.9121 (5)	0.0527 (8)	
H13A	0.2788	−0.0009	0.9696	0.063*	
H13B	0.1657	0.0070	0.8443	0.063*	
C14	0.4276 (3)	0.07773 (12)	0.9248 (5)	0.0538 (8)	
H14A	0.4784	0.0541	0.9824	0.065*	
H14B	0.4913	0.0965	0.8649	0.065*	

### Atomic displacement parameters (Å^2^)

**Table:**

	*U*^11^	*U*^22^	*U*^33^	*U*^12^	*U*^13^	*U*^23^
O1	0.0513 (12)	0.0591 (13)	0.0445 (12)	0.0059 (10)	0.0039 (11)	0.0162 (11)
N1	0.0433 (12)	0.0404 (11)	0.0415 (14)	0.0062 (11)	0.0031 (12)	0.0088 (12)
C1	0.0506 (17)	0.0527 (18)	0.091 (3)	0.0030 (16)	−0.006 (2)	−0.008 (2)
C2	0.0398 (14)	0.0402 (14)	0.061 (2)	0.0096 (12)	0.0003 (16)	0.0059 (15)
C3	0.0440 (15)	0.0423 (14)	0.0533 (19)	0.0089 (12)	−0.0061 (15)	0.0083 (14)
C4	0.0355 (13)	0.0356 (12)	0.0376 (15)	−0.0056 (10)	−0.0048 (12)	0.0012 (12)
C5	0.0320 (12)	0.0327 (12)	0.0316 (14)	−0.0013 (9)	0.0006 (11)	0.0011 (11)
C6	0.0393 (14)	0.0337 (12)	0.0495 (18)	−0.0075 (11)	−0.0055 (14)	0.0026 (13)
C7	0.0413 (15)	0.0443 (14)	0.0507 (18)	−0.0048 (12)	−0.0143 (15)	0.0036 (15)
C8	0.0570 (18)	0.0448 (15)	0.0375 (16)	0.0059 (13)	−0.0032 (15)	0.0103 (14)
C9	0.0437 (15)	0.0325 (12)	0.056 (2)	−0.0045 (11)	0.0028 (14)	0.0126 (14)
C10	0.0340 (12)	0.0351 (12)	0.0550 (18)	−0.0039 (10)	0.0027 (14)	0.0066 (14)
C11	0.0561 (18)	0.0492 (17)	0.0393 (18)	0.0108 (14)	0.0026 (15)	−0.0028 (13)
C12	0.0593 (19)	0.0477 (16)	0.050 (2)	0.0201 (14)	0.0100 (17)	−0.0022 (16)
C13	0.0624 (19)	0.0313 (13)	0.065 (2)	0.0051 (13)	−0.0091 (19)	−0.0010 (15)
C14	0.0365 (14)	0.0611 (18)	0.064 (2)	0.0086 (13)	0.0026 (16)	0.0153 (19)

### Geometric parameters (Å, °)

**Table:**

O1—C4	1.225 (4)	C8—C9	1.525 (4)
N1—C4	1.340 (4)	C8—H8A	0.9700
N1—C3	1.461 (3)	C8—H8B	0.9700
N1—H1A	0.86 (3)	C9—C13	1.529 (5)
C1—C2	1.170 (5)	C9—C10	1.541 (4)
C1—H1	0.9300	C9—H9	0.9800
C2—C3	1.466 (4)	C10—H10A	0.9700
C3—H3A	0.9700	C10—H10B	0.9700
C3—H3B	0.9700	C11—C12	1.529 (4)
C4—C5	1.526 (4)	C11—H11A	0.9700
C5—C11	1.538 (4)	C11—H11B	0.9700
C5—C10	1.539 (4)	C12—C13	1.526 (5)
C5—C6	1.540 (4)	C12—C14	1.527 (5)
C6—C7	1.531 (4)	C12—H12	0.9800
C6—H6A	0.9700	C13—H13A	0.9700
C6—H6B	0.9700	C13—H13B	0.9700
C7—C14	1.521 (5)	C14—H14A	0.9700
C7—C8	1.527 (4)	C14—H14B	0.9700
C7—H7	0.9800		
			
C4—N1—C3	121.0 (3)	H8A—C8—H8B	108.3
C4—N1—H1A	120.7 (19)	C8—C9—C13	110.0 (3)
C3—N1—H1A	115.4 (19)	C8—C9—C10	109.8 (2)
C2—C1—H1	180.0	C13—C9—C10	109.1 (3)
C1—C2—C3	175.9 (4)	C8—C9—H9	109.3
N1—C3—C2	110.7 (2)	C13—C9—H9	109.3
N1—C3—H3A	109.5	C10—C9—H9	109.3
C2—C3—H3A	109.5	C5—C10—C9	109.9 (2)
N1—C3—H3B	109.5	C5—C10—H10A	109.7
C2—C3—H3B	109.5	C9—C10—H10A	109.7
H3A—C3—H3B	108.1	C5—C10—H10B	109.7
O1—C4—N1	121.3 (3)	C9—C10—H10B	109.7
O1—C4—C5	121.5 (3)	H10A—C10—H10B	108.2
N1—C4—C5	117.2 (2)	C12—C11—C5	110.1 (3)
C4—C5—C11	110.4 (2)	C12—C11—H11A	109.6
C4—C5—C10	114.1 (2)	C5—C11—H11A	109.6
C11—C5—C10	108.5 (2)	C12—C11—H11B	109.6
C4—C5—C6	106.6 (2)	C5—C11—H11B	109.6
C11—C5—C6	108.7 (2)	H11A—C11—H11B	108.1
C10—C5—C6	108.5 (2)	C13—C12—C11	110.0 (3)
C7—C6—C5	110.5 (2)	C13—C12—C14	109.3 (3)
C7—C6—H6A	109.5	C11—C12—C14	109.5 (3)
C5—C6—H6A	109.5	C13—C12—H12	109.3
C7—C6—H6B	109.5	C11—C12—H12	109.3
C5—C6—H6B	109.5	C14—C12—H12	109.3
H6A—C6—H6B	108.1	C12—C13—C9	109.1 (2)
C14—C7—C8	109.8 (2)	C12—C13—H13A	109.9
C14—C7—C6	109.5 (3)	C9—C13—H13A	109.9
C8—C7—C6	109.0 (2)	C12—C13—H13B	109.9
C14—C7—H7	109.5	C9—C13—H13B	109.9
C8—C7—H7	109.5	H13A—C13—H13B	108.3
C6—C7—H7	109.5	C7—C14—C12	109.7 (2)
C9—C8—C7	109.3 (3)	C7—C14—H14A	109.7
C9—C8—H8A	109.8	C12—C14—H14A	109.7
C7—C8—H8A	109.8	C7—C14—H14B	109.7
C9—C8—H8B	109.8	C12—C14—H14B	109.7
C7—C8—H8B	109.8	H14A—C14—H14B	108.2
			
C4—N1—C3—C2	−82.6 (4)	C8—C9—C10—C5	59.7 (3)
C3—N1—C4—O1	−16.5 (4)	C13—C9—C10—C5	−60.9 (3)
C3—N1—C4—C5	160.2 (3)	C4—C5—C11—C12	175.3 (3)
O1—C4—C5—C11	−21.6 (4)	C10—C5—C11—C12	−59.0 (3)
N1—C4—C5—C11	161.8 (3)	C6—C5—C11—C12	58.8 (3)
O1—C4—C5—C10	−144.0 (3)	C5—C11—C12—C13	60.0 (4)
N1—C4—C5—C10	39.4 (3)	C5—C11—C12—C14	−60.2 (3)
O1—C4—C5—C6	96.2 (3)	C11—C12—C13—C9	−60.3 (4)
N1—C4—C5—C6	−80.4 (3)	C14—C12—C13—C9	60.0 (3)
C4—C5—C6—C7	−177.4 (2)	C8—C9—C13—C12	−60.1 (3)
C11—C5—C6—C7	−58.4 (3)	C10—C9—C13—C12	60.4 (3)
C10—C5—C6—C7	59.3 (3)	C8—C7—C14—C12	59.9 (3)
C5—C6—C7—C14	59.4 (3)	C6—C7—C14—C12	−59.8 (3)
C5—C6—C7—C8	−60.8 (3)	C13—C12—C14—C7	−60.2 (3)
C14—C7—C8—C9	−59.3 (3)	C11—C12—C14—C7	60.4 (3)
C6—C7—C8—C9	60.7 (3)	O1—C4—C3—C2	−87.7 (3)
C7—C8—C9—C13	59.5 (3)	C4—N1—C3—C2	−82.6 (4)
C7—C8—C9—C10	−60.5 (3)	O1—C4—N1—H1A	−176 (2)
C4—C5—C10—C9	−176.9 (2)	H1A—N1—C3—C2	78 (2)
C11—C5—C10—C9	59.6 (3)	O1—C4—C5—C6	96.2 (3)
C6—C5—C10—C9	−58.3 (3)		

### Hydrogen-bond geometry (Å, °)

**Table:**

*D*—H···*A*	*D*—H	H···*A*	*D*···*A*	*D*—H···*A*
N1—H1A···O1^i^	0.86 (3)	2.16 (4)	2.984 (4)	161 (3)
C1—H1···O1^ii^	0.93	2.41	3.188 (5)	141
C10—H10B···O1^i^	0.97	2.57	3.515 (4)	164
C3—H3A···X1^iii^	0.97	2.93	3.833	157
C3—H3B···X1^iv^	0.97	2.93	3.813	151
C6—H6A···X1^v^	0.97	2.81	3.689	151

Symmetry codes: (i) −*x*, *y*, *z*+1/2; (ii) −*x*+1/2, −*y*+1/2, *z*+1/2; (iii) *x*−1/2, *y*+1/2, *z*+1/2; (iv) −*x*, *y*, *z*−1/2; (v) *x*+1/2, *y*+1/2, *z*+1/2.
